# Prelimbic Cortical Stimulation with L-methionine Enhances Cognition through Hippocampal DNA Methylation and Neuroplasticity Mechanisms

**DOI:** 10.14336/AD.2022.0706

**Published:** 2023-02-01

**Authors:** Chi Him Poon, Yanzhi Liu, Sojeong Pak, Robert Chunhua Zhao, Luca Aquili, George Lim Tipoe, Gilberto Ka-Kit Leung, Ying-Shing Chan, Sungchil Yang, Man-Lung Fung, Ed Xuekui Wu, Lee Wei Lim

**Affiliations:** ^1^School of Biomedical Sciences, Li Ka Shing Faculty of Medicine, The University of Hong Kong, Hong Kong SAR, China.; ^2^Department of Neuroscience, City University of Hong Kong, Kowloon, Hong Kong, China.; ^3^School of Life Sciences, Shanghai University, Shanghai, China.; ^4^College of Science, Health, Engineering and Education, Discipline of Psychology, Murdoch University, Perth, Australia.; ^5^Department of Surgery, Li Ka Shing Faculty of Medicine, The University of Hong Kong, Hong Kong, China.; ^6^Department of Electrical and Electronic Engineering, The University of Hong Kong, Hong Kong, China.

**Keywords:** Neuroepigenetics, neuromodulation, DNA methylation, DNA methyltransferase, age-related cognitive decline

## Abstract

Declining global DNA methylation and cognitive impairment are reported to occur in the normal aging process. It is not known if DNA methylation plays a role in the efficacy of memory-enhancing therapies. In this study, aged animals were administered prelimbic cortical deep brain stimulation (PrL DBS) and/or L-methionine (MET) treatment. We found that PrL DBS and MET (MET-PrL DBS) co-administration resulted in hippocampal-dependent spatial memory enhancements in aged animals. Molecular data suggested MET-PrL DBS induced DNA methyltransferase DNMT3a-dependent methylation, robust synergistic upregulation of neuroplasticity-related genes, and simultaneous inhibition of the memory-suppressing gene calcineurin in the hippocampus. We further found that MET-PrL DBS also activated the PKA-CaMKIIα-BDNF pathway, increased hippocampal neurogenesis, and enhanced dopaminergic and serotonergic neurotransmission. We next inhibited the activity of DNA methyltransferase (DNMT) by RG108 infusion in the hippocampus of young animals to establish a causal relationship between DNMT activity and the effects of PrL DBS. Hippocampal DNMT inhibition in young animals was sufficient to recapitulate the behavioral deficits observed in aged animals and abolished the memory-enhancing and molecular effects of PrL DBS. Our findings implicate hippocampal DNMT as a therapeutic target for PrL DBS and pave way for the potential use of non-invasive neuromodulation modalities against dementia.

Dementia is a progressive condition that commonly occurs in the older population. The development of effective dementia treatments is challenging, as demonstrated by the frequent failure to validate the therapeutic effects of drug interventions in clinical trials [1, 2]. Because of the multifactorial nature of aging, treatments for dementia that target specific age-related disease pathways through pharmacological means may result in limited therapeutic effectiveness [2-5]. Deep brain stimulation (DBS) presents a novel modality for alleviating cognitive deterioration by stimulating neural circuits that underlie cognitive ability, and by reactivating processes such as hippocampal neurogenesis and plasticity that are impaired in aging [6-10]. Recent studies have identified the DNA methylation machinery and the methylation patterns of specific gene loci can be modulated by neuronal stimulation, which suggests DNA methylation may be a pivotal factor involved in the therapeutic efficacy of neuromodulation techniques [11-13]. These findings support the potential use of DBS to ameliorate DNA methylation-related deficits such as hippocampal DNA hypomethylation caused by attenuated DNA methylation machinery associated with aging [14, 15]. Although such deficits can also be restored by methyl supplements, their effects on cognition remain debatable [16]. The prelimbic cortex (PrL) has extensive anatomical connectivity and is a region associated with widespread neuroplasticity and neurotransmitter changes in the hippocampus [6, 8, 17, 18]. This study aimed to investigate the cognitive-enhancing effects of protracted administration of L-methionine (MET) combined with DBS in the PrL (PrL DBS) in an aged animal model, and to identify the molecular targets necessary for these effects. Elucidating how MET and PrL DBS elicit their therapeutic effects on ameliorating DNA hypomethylation and cognitive deficits in aged animal models may yield translational insights.

We hypothesized that DNA methylation is a key molecular component underlying the efficacy of DBS in modulating memory-related processes [19, 20]. We found that chronic PrL DBS with protracted MET administration specifically improved hippocampus-dependent memory performance in aged animals. We further demonstrated that the MET-PrL DBS treatment synergistically enhanced hippocampal DNA methylation levels and modified synaptic plasticity- and cognition-related genes and proteins. Moreover, we showed that inducing hippocampal DNA hypomethylation in young animals was sufficient to recapitulate the anxiety and cognitive deficits observed in aged animals and revealed a partial causal role of DNA methylation in the effects of PrL DBS on memory. In summary, we identified that hippocampal DNA methylation is a critical factor for the cognitive-enhancing effects of PrL DBS. Our findings support the targeting of epigenetic regulation in the hippocampus as a potential therapeutic strategy for memory enhancement.

## MATERIALS AND METHODS

### Subjects

**Figure 1.**
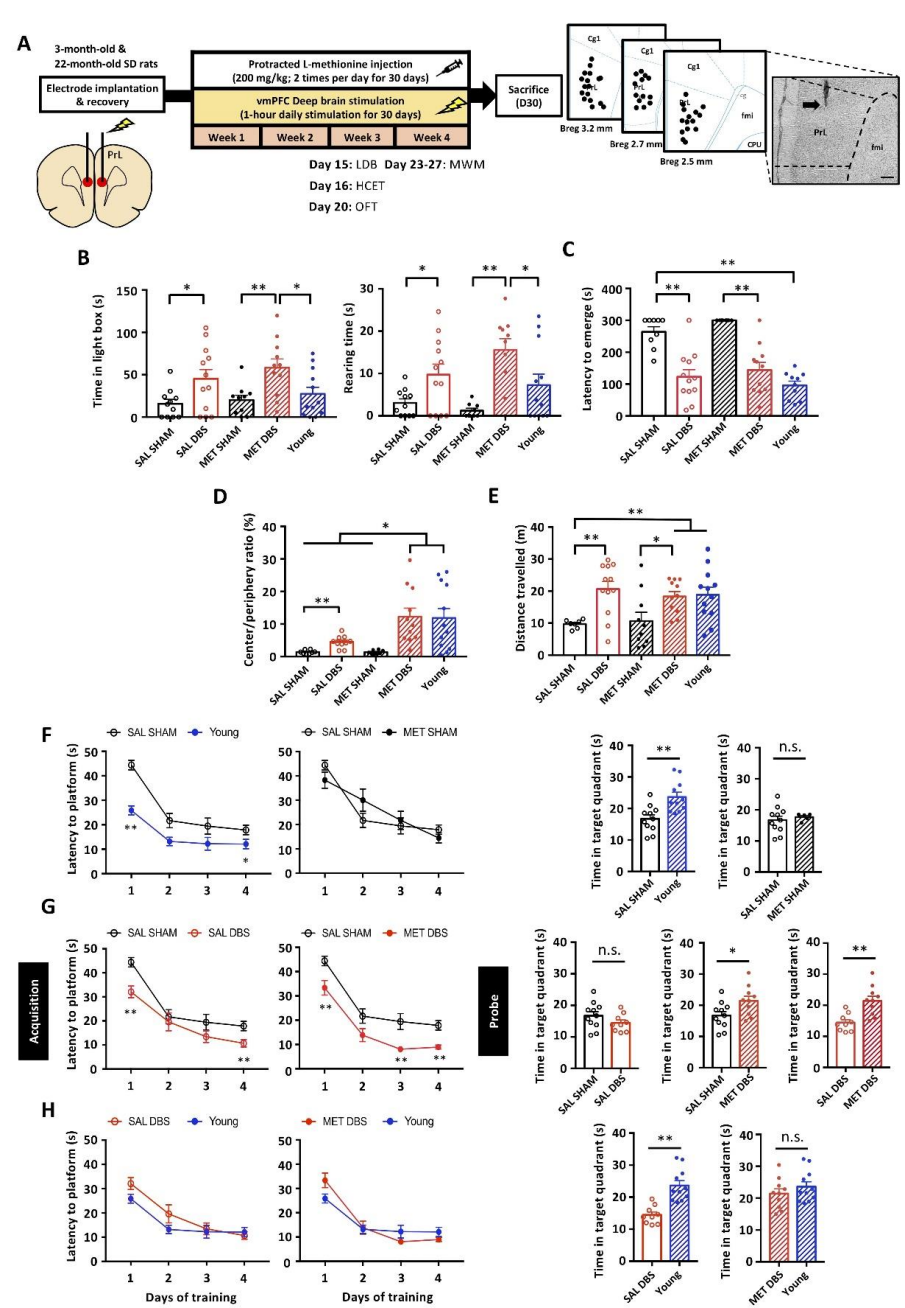
PrL DBS alone is sufficient to reduce anxiety and improve spatial learning in aged animals, whereas MET-DBS enhances both spatial learning and memory. Schematic representation of the experimental timeline and behavioral tests for anxiety and memory in aged (n=43) and naïve young (n=12) animals (SAL-SHAM: n=11; SAL-DBS: n=11; MET-SHAM: n=10; MET-DBS: n=11; naïve young group: n=12). Photomicrograph confirming electrode localization in the PrL. Scale bar: 200 µm (A). In the LDB test, PrL DBS increased the time spent (p<0.001) and rearing behavior (p<0.001) in the light box (B). In HCET, PrL DBS reduced the latency to emerge from the home cage. Two-way ANOVA revealed a significant effect of stimulation (p<0.001) (C). In the OFT, PrL DBS increased the time spent in the center of the open field (Stimulation, p<0.001; Treatment, p=0.023; Stimulation x Treatment, p=0.022) (D) and locomotor activity (Stimulation, p<0.001) (E). In the MWM test, all animals were trained to locate the hidden platform for 4 days before the probe test without the platform 24 h after the last training session. Three-way repeated measures ANOVA indicated significant main effects of Stimulation x Treatment x Time (p=0.009) and Stimulation (p<0.001). SAL-SHAM animals showed increased latency to locate the hidden platform during training (p<0.001) and reduced time spent in the target quadrant during the probe test compared to young animals (p<0.01), indicating age-related cognitive decline. No differences were observed in the escape latency during training and time spent in the target quadrant during the probe test between SAL-SHAM and MET-SHAM animals (F). Both SAL-DBS and MET-DBS animals showed reduced escape latency during training compared to SAL-SHAM animals. MET-DBS animals showed more time spent in the target quadrant during the probe test than SAL-SHAM animals (p<0.05), whereas no difference in the time spent in the target quadrant was observed between SAL-SHAM and SAL-DBS animals (G). No difference in spatial learning was observed between DBS-treated animals and young animals. However, SAL-DBS animals spent significantly less time in the target quadrant during the probe test compared to young animals (p<0.01), whereas MET-DBS animals spent a comparable time in the target quadrant to that of young animals (H). *, p<0.05; **, p<0.01; n.s., not significant. Data presented as mean ± s.e.m.

**Figure 2.**
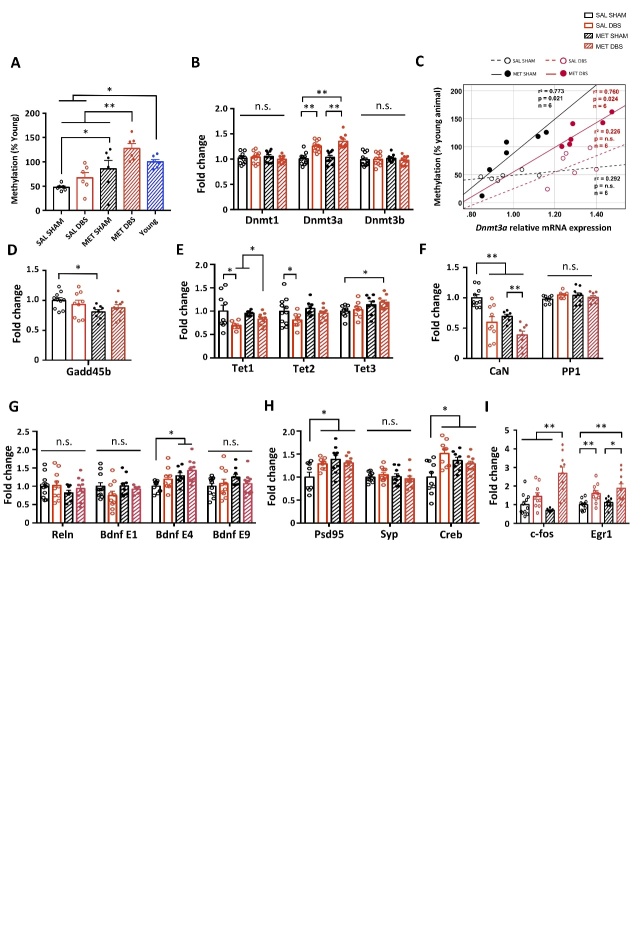
Protracted MET treatment and PrL DBS alters DNA methylation machinery and neuroplasticity-associated gene expressions. Statistical analysis with two-way ANOVA revealed that SAL-SHAM animals showed decreased global DNA methylation in the hippocampus compared to young animals, whereas protracted MET treatment administered alone or in combination with PrL DBS restored DNA methylation levels in aged animals (Stimulation, p = 0.016; Treatment, p < 0.001). All groups: n=6. (A). Quantitative reverse-transcription PCR analysis of *Dnmt1* (p > 0.221), *Dnmt3a* (p < 0.001), *Dnmt3b* (p > 0.493) (B). Scatter plot displaying significant correlations between DNA methylation level and *Dnmt3a* mRNA expression in MET-SHAM and MET-DBS animals, indicating the upregulation of Dnmt3a increased global DNA methylation in the hippocampus of aged animals (C). Quantitative reverse-transcription PCR analysis of *Gadd45b* (Treatment, p = 0.026) (D), *Tet1* (Stimulation, p = 0.008), *Tet2* (Stimulation, p = 0.039), *Tet3* (Treatment, p = 0.012) (E), *CaN* (Treatment, p < 0.001; Stimulation, p <0.001), *Pp1* (p > 0.119) (F), *Reln* (p > 0.201), *Bdnf* exon I (p > 0.117), *Bdnf* exon IV (Treatment, p = 0.006), *Bdnf* exon IX (p > 0.201) (G), *Psd95* (Treatment, p = 0.02; Treatment x Stimulation, p = 0.033), *Syp* (p > 0.364) (H), *c-fos* (Treatment, p = 0.045; Stimulation, p < 0.001; Treatment x Stimulation, p = 0.002), *Egr1* (Stimulation, p < 0.001). SAL-SHAM: n=11; SAL-DBS: n=11; MET-SHAM: n=10; MET-DBS: n=11. (I). *, p<0.05; **, p<0.01; n.s., not significant. Data presented as mean ± s.e.m.

Male Sprague-Dawley rats [3 months old, n= 66 (Fig. 1, 5, 6, 7A-I); 18 months old, n=26 (Fig. 4A-D); 23 months old, n= 43 (Fig. 1, 2, 3 4E-M)] were individually housed and maintained in a 12 h light:dark cycle (lights on at 22:00) with access to food and water *ad libitum* at our on-site Association for Assessment and Accreditation of Laboratory Animal Care (AAALAS)-accredited facility. The housing environment was maintained at a temperature of 21±1°C and humidity of 60%-65%. All the experimental procedures and tests were conducted during the dark cycle. Behavioral and molecular charac-terizations of the animals were performed and analyzed by researchers blinded to the animal treatments. The study was approved by the Committee on the Use of Live Animals in Teaching and Research of The University of Hong Kong (Ref: 4549-17).

### Surgery and Deep Brain Stimulation

Surgery was performed as previously described [6, 17]. In brief, rats were anesthetized with 5% isoflurane and maintained in the plane of anesthesia with 2%-3% isoflurane before being placed in a stereotactic frame. Gold-plated electrodes with platinum-iridium cores (Synergy Engineering Pte Ltd, Singapore) were implanted bilaterally in the PrL (AP: +2.7 mm; ML: ±0.6 mm; DV: -3.0 mm) based on the Paxinos and Watson rat brain atlas [21]. For young animals, a bilateral cannula was implanted in addition to the bipolar electrodes. The animals receiving bilateral cannulation were implanted with a guide cannula (RWD Life Science) in the dorsal hippocampus (dHPC) (AP: -4 mm; ML: ±2 mm; DV: -2.4 mm) [22]. The electrodes and guide cannula were fixed on the skull by dental cement. Animals were allowed to recover for 2 weeks. After recovery, animals were randomly allocated into four groups: 1) SAL-SHAM: saline-treated sham; n=11, 2) SAL-DBS: saline-treated DBS; n=11, 3) MET-SHAM: methionine-treated sham; n=10, and 4) MET-DBS: methionine-treated DBS; n=11. A digital stimulator (Model 3800 MultiStim: 8-channel Stimulator; A-M Systems, Carlsborg, USA) and eight stimulus isolators (Model 3820; A-M Systems) were used to deliver the electrical stimuli to rats. Animals in both DBS groups received 1 h of electrical stimulation daily for 4 weeks. Based on previously established parameters used in the cognitive assessment of DBS in rodents [6, 8], biphasic rectangular pulses (100 Hz; 200 μA; 100 μs pulse width) were applied. Animals in the sham groups were treated in a similar manner, but no electrical stimulation was administered. Behavioral testing was conducted starting from day 15 of DBS. During the period of behavioral testing, animals were stimulated for 30 min prior to the behavioral testing. After the behavioral task, animals were again stimulated for another 30 min to maintain 1 h of stimulation per day, as previously described [6, 8].

### Drug treatment

Aged rats received a subcutaneous injection of saline (0.9% NaCl) or methionine (200 mg/kg; Sigma-Aldrich, St. Louis, Missouri, USA) twice a day (12-h interval) for 30 days. This dosage was previously shown to induce global DNA hypermethylation [23]. For the RG108 experiment, bilaterally cannulated young rats (ACSF-SHAM: n=12; ACSF-DBS: n=12; RG108-SHAM: n=14; RG108-DBS: n=12) were infused with either RG108 (Axon Medchem) or artificial cerebral spinal fluid (ACSF) for 30 days at a dosage previously shown to reduce DNA methylation [24]. RG108 in 20% DMSO was diluted to 200 ng/µL in ACSF. Drugs (2 µL) were infused into the dHPC using a Hamilton syringe connected to the internal cannula through polyethylene tubing (Protech international, Texas, USA) at a rate of 0.5 µL/min over 4 min. The internal cannula was left inside the guide cannula for an additional 3 min to ensure proper diffusion of the infused drug.

### BrdU injection

The administration of BrdU was performed as previously described [6]. Briefly, during the first week of DBS, all animals were intraperitoneally injected with 5-bromo-2’-deoxyuridine (BrdU, Cat# B5002, Sigma, Missouri, USA) at 150 mg/kg per injection every 2 h three times daily on days 1, 3, 5, and 7 [6]. The first dose of BrdU was administered 30 min before the 1-h stimulation, with subsequent injections performed at 2-h intervals.

### Light-dark box (LDB) test

The LDB test was performed following previously established protocol with minor modifications [25]. An arena was separated into a clear Plexiglas light chamber (40 x 40 x 40 cm) and black chamber (40 x 40 x 40 cm) was covered with a black lid. Access in between the two chambers was allowed through a small opening. The animal was placed in the dark chamber and allowed to freely explore inside the arena for 5 min. The experiment was recorded with an overhead camera connected to an ANY-maze system to score the time spent and frequency of rearing behavior in the light chamber.

### Home cage emergence test (HCET)

The HCET test was performed as published with minor modifications [17, 26, 27]. The test was performed in the animals’ home cage with the lid removed. The home cage and a novel cage were placed on a platform. A metal grid was positioned over the edge of the two cages to facilitate the movement of the animal across the two cages. The animal was left in the home cage for 5 min. The experiment was recorded with an overhead camera to score the latency to emerge from the home cage. The time required for the animal to leave the home cage and climb onto the grid was measured. The animal must place all their limbs on the grid to be scored as an exit from the home cage. Animals that failed to leave the home cage within 5 min were given a score of 300 s.

### Open field test (OFT)

The OFT test was performed as previously described [8, 28]. Briefly, the animal was allowed to explore freely for 5 min in the open arena of a square Plexiglas box (100 x 100 x 40 cm) with an open top and dark floor. The experiment was recorded with an overhead camera to track the animal’s movement.

### Morris water maze (MWM)

The MWM test was performed as previously described with minor modifications [6, 8]. Briefly, the test was performed in a black circular pool (150 cm diameter) filled with water maintained at a temperature of 25±1°C. The test assesses the animal’s ability to locate the submerged transparent circular escape platform (10 cm Diameter) placed 2 cm below water surface and 35 cm from the edge of the pool. In the acquisition phase, the animals were trained to locate the submerged platform from a randomized starting position equidistant from the platform in four trials per day for 4 consecutive days. Each animal was allowed 1 min to find the submerged platform. If the animal failed to locate the submerged platform within 1 min, it was gently guided to the platform and was allowed to stay on the platform for 15 s. The probe phase was performed 24 h after the last training session. During the probe phase, the platform was removed, and each animal was allowed 1 min to explore the pool. The searching time of animals inside each quadrant was recorded.

### Global DNA methylation quantification

This assay was performed as previously described [23]. Briefly, Epigentek’s MethylFlash™ Global DNA methylation ELISA Easy kit (Cat# P-1030) was used according to the manufacturer’s instructions. This approach is suitable, sensitive, and effective for the small amounts of starting DNA material from our hippocampal tissue samples. Absorbance was colorimetrically quantified and the total DNA methylation level was estimated by comparing the raw data with the calibration curve generated using methylated DNA standards.

**Table 1:** PCR primers.

Target gene	Sequence (5’ - 3’)
Dnmt1	Forward: AGAACGGAACACTCTCTCTCACTCA Reverse: AAGCTTCAATCATGGTCTCACTGTC
Dnmt3a	Forward: CCAAGTGGACCGCTACATC Reverse: ACTCCTGGATATGCTTCTGTG
Dnmt3b	Forward: ACAGCGGAGGATGCCAAGC Reverse: TCTTCCAGGTCAGGATCAGTAGTG
Tet1	Forward: CATGGAGACTAGGTATGGCCAGAAGG Reverse: CTTCTTCTAATCACCCACTTGGCGAC
Tet2	Forward: GAACAATCATGGAAGAAAGGTTTGGAGA Reverse: GTACTTGACCTCCGATACACCCATTTAG
Tet3	Forward: GGGAACTCATGGAGGATCGGTATGGAG Reverse: GTGTGTGTCTTCGGATCACCCACTTG
Gadd45b	Forward: CTCCTGGTCACGAACTGTCA Reverse: GGGTAGGGTAGCCTTTGAGG
CaN (Ppp3ca)	Forward: GCAGGCTGGAAGAAAGTGTC Reverse: AAGGCCCACAAATACAGCAC
Pp1 (Ppp1cc)	Forward: AGTATCATCCAACGGCTGCT Reverse: CCCGTGGATGTCACCACATA
Reln	Forward: AAACTACAGCGGGTGGAACC Reverse: ATTTGAGGCATGACGGACCTATAT
Bdnf exon I	Forward: CAGGGCAGTTGGACAGTCAT Reverse: TACGCAAACGCCCTCATTCT
Bdnf exon IV	Forward: ACTGAAGGCGTGCGAGTATT Reverse: TGGTGGCCGATATGTACTCC
Bdnf exon IX	Forward: GAGAAGAGTGATGACCATCCT Reverse: TCACGTGCTCAAAAGTGTCAG
Psd95	Forward: GACATCACAACCTCGTATTCTC Reverse: AAGATGCCTTCACCATCCTC
Syn	Forward: CTTTCTGGCTACAGCCGTGTTCG Reverse: GTTCCCTGTCTGGCGGCACATG
Creb	Forward: TGCCCCTGGAGTTGTTATGG Reverse: TCCAGTCCATTTTCCACCACATA
Egr1	Forward: GACCACCTTACCACCCACATCC Reverse: GCCTCTTGCGTTCATCACTCCT
c-fos	Forward: CCGACTCCTTCTCCAGCAT Reverse: TCACCGTGGGGATAAAGTTG
Tubulin, beta 4	Forward: AGCAACATGAATGACCTGGTG Reverse: GCTTTCCCTAACCTGCTTGG

### Reverse transcription real-time PCR

This assay was performed as previously described [6, 8, 29, 30]. Animals were decapitated on day 30 after receiving 1-h electrical stimulation. The dHPC was dissected and total RNA was extracted using TRIzol reagent (Cat# TR118, Molecular Research Center Inc., Ohio, USA). RNA concentration and purity were determined using a NanoDrop spectrophotometer (Thermo Fisher Scientific, USA) through calculating the ratio of optical density at wavelengths between 260nm and 280nm. 7µl of RNA (600-800 ng in total) was used for cDNA synthesis using a PrimeScript™ RT reagent kit with gDNA eraser (Cat# RR047B, Takara Bio USA, California, USA) according to the instructions from manufacturer. Briefly, the RNA mixture was incubated with gDNA eraser and eraser buffer at 42°C for 2 min, followed by incubation in reverse transcription buffer, enzyme and primer mix at 37°C for 15 min, and finally with incubation at 85°C for 5 s to produce the final cDNA synthesis mixture. Efficiency of primers was validated using the diluted cDNA series measured between 90-110%. Real-time PCR was performed on a Roche LightCycler 480 II (Roche Diagnostics). Relative gene expressions were analyzed using the 2^-DDCT^ method relative to the expression of SAL-SHAM group (for aged animal experiments) or ACSF-SHAM group (for young animal experiments) [29, 30] (Table 1).

### Western blotting

The procedure was performed as previously described [29, 31]. Briefly, the dHCP was rapidly gross-dissected followed by homogenization in cold lysis buffer (0.15 M NaCl, 0.05 M Tris-HCl, 1% Triton x100, 0.1% sodium dodecyl sulfate, 0.5% sodium deoxycholate, 1 mM EDTA) containing a protease and phosphatase inhibitor cocktail (Cat# P178442, Thermo Fisher Scientific). Homogenates were incubated at 4°C for 1.5 h and centrifuged at 12000 g for 5 min. Protein concentrations were determined by Bradford assay (Cat# 5000205, Bio-rad Laboratories). Equal amounts of total protein (20-30 µg) were separated on 10% SDS-PAGE gels and then transferred to PVDF membranes. The membrane was blocked with 5% milk or BSA in TBS at room temperature for 1 h and then incubated with primary antibodies overnight at 4°C. Antibodies against PSD95 (1:1000, Cat# ab18258, Abcam), synaptophysin (1:1000, Cat# 5461, Cell Signaling Technology), CREB (1:1000, Cat# 9197, Cell Signaling Technology), pCREB (1:1000, Cat# 9198, Cell Signaling Technology), BDNF (1:1000, Cat# NB100-98682, Novus Biologicals), PKA C-α (1:1000, Cat# 4782, Cell Signaling Technology), pPKA C^Thr197^ (1:1000, Cat# 4781, Cell Signaling Technology), ERK1/2 (1:1000, Cat# 9102, Cell Signaling Technology), pERK1/2^T202/Y204^ (1:1000, Cat# 9101, Cell Signaling Technology), CaMKIIα (1:1000, Cat# 3362, Cell Signaling Technology), pCaMKIIα^Thr286^ (1:1000, Cat# 12716, Cell Signaling Technology), MeCP2 (1:1000, Cat# NB600-1101, Novus Biologicals), pMeCP2^S421^ (1:1000, Cat# NBP2-29524, Novus Biologicals), DNMT3a (1:1000, Cat# 3598, Cell Signaling Technology), Calcineurin A (1:1000, Cat# 2614, Cell Signaling Technology) and GAPDH (1:1000, Cat# 2118, Cell Signaling Technology) were used. The membrane was washed and subsequently probed with HRP-conjugated goat anti-rabbit antibody (1:2000, Cat# 31460, Thermo Fisher) for 1 h. Immunoblot signals were visualized by Clarity Western ECL Substrate (Cat# 1705061, Bio-rad) and detected by the ChemiDoc Imaging system (Bio-rad).

### Electrophysiology

The procedures for brain preparation and electro-physiology were performed based on previous established protocols [32, 33]. Briefly, Male Sprague-Dawley rats were anesthetized with inhalable isoflurane followed by an intraperitoneal injection of sodium pentobarbital. The brains were quickly removed and placed into chilled (4°C) oxygenated (5% CO_2_ and 95% O_2_) slicing medium containing 212 mM sucrose, 5 mM KCl, 1.23 mM NaH_2_PO_4_, 26 mM NaHCO_3_, 11 mM glucose, 1.5 mM MgCl_2_ and 2.5 mM CaCl_2_. Brain slices (400 μm) of the dHCP (Bregma -2.64 to -3.84 mm) were cut coronally. Slices were transferred to a holding chamber containing oxygenated artificial cerebrospinal fluid (ACSF; 24 mM NaCl, 4 mM KCl, 1.23 mM NaH_2_PO_4_, 26 mM NaHCO_3_, 10 mM glucose, 1.5 mM MgCl_2_ and 2.5 mM CaCl_2_). After 1 h of recovery at 35°C and at least 1 h at room temperature (20°C), the hippocampal slices were transferred from the recovery beakers to the recording chambers, where they were submerged in ACSF (125 mM NaCl, 2.5 mM KCl, 1.3 mM NaH_2_PO_4_, 25 mM NaHCO_3_, 25 mM glucose, 1 mM MgCl_2_ and 2 mM CaCl_2_; 32°C) delivered at a flow rate of 1.5 mL/min and bubbled with a mixture of 95% O_2_ and 5% CO_2_. Stimulating electrodes (concentric bipolar microelectrode, FHC, USA) were positioned in the medial perforant path (MPP) in the brain slices and local field potentials (LFPs) were evoked (constant current, 1 ms duration, repeated at 30-s intervals). The LFPs were recorded from the middle molecular layer (MML) of dentate gyrus (DG) using glass electrodes containing ACSF. Long-term synaptic potentiation (LTP) was induced by four trains of a tetanus stimuli (high frequency stimulation [HFS]; 100 Hz in 1 s, 1 ms duration with 9 ms interpulse interval, four times at 15-s intervals) after acquiring > 20 min of baseline recordings. In all the experiments, picrotoxin (100 µM; Sigma) was applied to the perfusate 15-20 min before the recordings were acquired. The peak amplitudes of the LFPs were measured and expressed relative to the normalized pre-conditioning baselines. Before the LTP was induced, the input/output (I/O) curves and paired-pulse responses were identified.

### Mass spectrometry

The MS analysis was performed as previously described [8, 22]. Briefly, tissues were homogenized, and metabolites extracted in 1.5 mL of methanol/MilliQ water (80%, v/v) with 0.1 mg norvaline as the internal standard. Homogenization of tissue was performed on ice by 10 cycles of sonication at 10 microns at 20- and 10-s pause times. Next, 750 μL MilliQ water was added, and the tube was vortexed for 30 s, followed by 1200 μL chloroform and further vortexed. After 15 min of agitation, the sample was centrifuged for 5 min at 10,000 *g*. The polar phase was isolated, and the dried residue was redissolved and derivatized for 2 h at 37°C in 70 μL N-Methyl-N-trimethylsilyltrifluoroacetamide (MSTFA) and 1% trimethylsilyl chloride (TMCS). A sample (0.2 μL) was analyzed by GC-MS and the remaining sample was dried under vacuum. Acquisition of the GC/MS spectra was done in SCAN and MRM mode on an Agilent 7890B GC and Agilent 7010 Triple Quadrupole Mass Spectrometer system (Agilent, CA, USA). The sample was separated in an Agilent DB-5MS capillary column (30 m x 0.25 mm ID, 0.25-μm film thickness) with a constant flow rate of 1 mL min^-1^. The GC oven program started at 60°C (holding time 1 min) and increased at 10°C min^-1^ to 120°C, then at 3°C min^-1^ to 150°C, followed by 10°C min^-1^ to 200°C and finally 30°C min^-1^ to 280°C (holding time 5 min). Inlet temperature and transfer line temperature were 250°C and 280°C, respectively. During the run, characteristic quantifier and qualifier transitions were monitored in MRM mode and mass spectra were acquired from m/z 50 to 500 in SCAN mode.

Data analysis was performed using the Agilent MassHunter Workstation Quantitative Analysis Software. Linear calibration curves for each analyte were generated by plotting the peak area ratio of external/internal standard against the standard concentration at different concentration levels. Analytes were confirmed by comparing the retention time and ratio of characteristic transitions between the sample and standard. Dopamine (DA), serotonin (5-HT), gamma-aminobutyric acid (GABA), glutamic acid (Glu), 3,4-dihydroxyphenylacetic acid (DOPAC), homovanillic acid (HVA), 5-hydroxyindole acetic acid (5-HIAA), and norepinephrine (NE) were measured with norvaline as the internal standard. Due to the low amount of neurotransmitters, samples are pooled (three samples pooled into one run and results were obtained from 3 biological replicates) for MS experiment.

### Flow cytometry

Fluorescence-activated cell sorting (FACS) acquisition and analyses were performed as previously described [34]. Briefly, sample preparation was performed by using a APC BrdU Flow kit (Cat# 552598, BD Pharmingen™) according to the manufacturer’s instructions. Rat hippocampal tissues were fixed in 4% PFA for 30 min, followed by mincing on ice and incubation in 4% PFA for 10 min. Tissues were then washed in PBS-T before enzymatic digestion by StemPro™ Accutase™ Cell Dissociation Reagent (Cat# A1110501, Thermo Fisher). Cells were collected, fixed, and permeabilized with BD Cytofix/Cytoperm Buffer. Incorporated BrdU was revealed by incubation with DNase for 1 h at 37°C. Cells were washed with BD Perm/Wash Buffer and stained with fluorochrome-conjugated anti-BrdU (Cat# 552598, APC, BD Pharmingen), anti-NeuN (Cat# MAB377x, Alexa Fluor 488, Millipore), and anti-GFAP (Cat# 561483, PE, BD Pharmingen) antibodies. Single antibody-stained samples were first analyzed to optimize the laser intensity and the necessary compensation to eliminate fluorescence leakage and background signals before the all-stained samples were screened. Analysis of the 20000 DAPI^+^ events was performed on a NovoCyte Quanteon Flow Cytometer using NovoExpress software.

### Statistics

Data analyses were conducted in IBM SPSS Statistics 25. All data were presented as the mean ± S.E.M. For experiments on aged animals, behavioral and molecular test results were analyzed by two-way analysis of variance (ANOVA) followed by orthogonal planned comparison for subsequent multiple comparisons as previously described [35], except MWM training data, which was analyzed by three-way repeated measures ANOVA followed by orthogonal planned comparison. For experiments on young animals with RG108, the results were analyzed by one-way or two-way ANOVA, followed by orthogonal planned comparison. Multiple comparisons were conducted based on the groups that showed significant main effects observed from ANOVA. Results of all multiple comparison were reported based on the homogeneity of variance derived from Levene’s test and normality derived from Shapiro-wilk test. Non-parametric of Kruskal-Wallis test was used for not normally distributed data, as appropriate. Pearson correlation coefficients with Bonferroni correction were calculated to assess the associations among behavioral parameters and gene and protein expressions related to hippocampal neuroplasticity and neurogenesis. For electrophysiology, results were analyzed by two-way repeated measures ANOVA followed by Bonferroni-corrected pairwise comparison. For MS experiment, the test results were analyzed by two-way ANOVA (aged animal) followed by orthogonal planned comparison based on sample homogeneity, or non-parametric Kruskal-Wallis test (young animals) followed by pairwise comparison based on the main effects found. Significance was defined as p<0.05.

## RESULTS

 

### PrL DBS enhances anxiolytic behavior in aged animals

Age-related anxiety has previously been shown in animal models [26]. We administered PrL DBS or Sham DBS with MET or saline (SAL) in aged rats under previously established parameters, and then studied the antidepressant and cognition-enhancing effects [6, 8, 17] (Fig. 1A). In the light-dark box (LDB) test, DBS-treated rats spent significantly more time and showed more rearing behaviors in the light box compared to Sham-treated rats regardless of MET/SAL treatment, which indicates PrL DBS induced anxiolytic effects (Fig. 1B). This finding was also replicated in the home cage emergence test (HCET), in which DBS rats displayed significantly reduced latency to emerge from the home cage regardless of MET/SAL treatment. Notably, DBS rats displayed a remarkably reduced latency level comparable to that of naïve young rats (Fig. 1C). We further assessed the effects of MET and PrL DBS on anxiety and locomotor function in the open field test (OFT). Although DBS rats displayed an enhanced preference for the center region regardless of MET/SAL, we observed even stronger center preference in MET-DBS rats comparable to that of naïve young rats (Fig. 1D). Similarly, MET-DBS rats showed improvements in the distance traveled in the open field. These findings demonstrated that while PrL DBS enhanced locomotor function, its combined administration with MET can induce anxiolytic effects in aged animals (Fig. 1E), which suggest the potential synergistic effects of MET and PrL DBS on anxiety.

### MET modulates the cognitive-enhancing effects of PrL DBS in aged animals

To determine whether MET and PrL DBS can improve spatial cognition, rats were trained for 4 days in the Morris water maze (MWM) before the memory probe test on day 5. We verified the aged animals (SAL-SHAM animals) had spatial learning and memory deficits, as demonstrated by the increased time to locate the hidden platform during training and significantly less time spent in the target quadrant during the memory probe test compared to naïve young animals. Treatment with MET alone did not alter the performance of the aged animals, as demonstrated by similar learning and retrieval performances between SAL-SHAM and MET-SHAM animals (Fig. 1F). Notably, PrL DBS significantly improved spatial learning regardless of MET/SAL treatment. Although no significant difference was found between SAL-DBS and MET-DBS groups during the acquisition phase, interestingly, PrL DBS with MET increased the time spent in the target quadrant compared with SAL-SHAM and SAL-DBS groups, indicating a more robust rescue of the spatial cognition deficits by the co-administration of PrL DBS and MET (Fig. 1G). This dual treatment paradigm improved both spatial learning and spatial memory performance in aged rats to a level comparable to that of naïve young rats (Fig. 1H). These results demonstrate that MET treatment can facilitate the cognition-enhancing effects of PrL DBS.

### MET and PrL DBS alter the expressions of genes involved in DNA methylation/demethylation in memory functions

Previous studies demonstrated that DNA methylation/demethylation influences synaptic plasticity and neurogenesis [36-41]. As aged animals are reported to have global DNA hypomethylation [14, 40], we hypothesized that increasing DNA methylation by MET and PrL DBS would rescue the neuroplasticity dysfunction during the aging process via DNA methylation-related mechanisms. After the behavioral screening, the dorsal hippocampus was harvested for downstream molecular assays. Compared to young animals, aged animals exhibited hippocampal global DNA hypomethylation, which could be rescued by the MET treatment. Previous work also showed that neuronal activity is a potent modulator of DNA methylation [42]. We found that PrL DBS alone could increase the DNA methylation level by up to 41%, although this was not statistically significant. However, MET-DBS animals showed a ~2.6-fold increase in the DNA methylation level compared to SAL-SHAM animals, demonstrating a robust potentiation effect from the combined treatments (Fig. 2A). We next examined DNMT levels in aged animals and determined whether it was involved in restoring DNA methylation by MET and/or PrL DBS. We found that PrL DBS, regardless of MET, specifically increased the level of *Dnmt3a* transcripts in the hippocampus of aged rats (Fig, 2B). We performed a correlational study to examine the relationship between *Dnmt3a* mRNA expression and DNA methylation level. We found a significant positive correlation between *Dnmt3a* mRNA expression and level of DNA methylation induced by MET alone and when combined with PrL DBS, but not by PrL DBS alone (Fig. 2C). These results confirmed our aged animal model exhibited global DNA hypomethylation, which could be rescued by MET and more strongly restored by MET combined with PrL DBS possibly via the activation of *Dnmt3a*.

To understand the molecular mechanism of MET and PrL DBS in the hippocampus, we analyzed hippocampal transcript levels of DNA demethylases, including growth arrest and DNA damage inducible beta (*Gadd45b)* and ten-eleven translocation methylcytosine deoxygenases (*Tet*s). We found that MET and PrL DBS had inhibitory effects on the expression levels of the respective DNA demethylases in the hippocampus, with the exception of *Tet3*. The MET treatment reduced *Gadd45b* mRNA (Fig. 2D) and resulted in enhanced *Tet3* expression levels, whereas PrL DBS attenuated *Tet1* and *Tet2* expression levels (Fig. 2E). Multiple comparisons showed the MET-DBS animals had significantly increased *Tet3* expression levels compared to SAL-SHAM animals, indicating PrL DBS may partially facilitate the increase of *Tet3* expression. Given that the Dnmts and DNA demethylases were associated with genes induced by neuronal activity [43, 44], we next assessed the expression levels of several candidate genes previously reported to either facilitate or suppress memory formation, including calcineurin (*CaN*) and protein phosphatase 1 (*Pp1*) that are thought to serve as memory constraints [45, 46]. Both MET and PrL DBS individually and synergistically reduced *CaN* transcript levels, whereas no significant differences were observed for *Pp1* among all groups (Fig. 2F). We also assessed the transcript levels of genes involved in neuroplasticity, including reelin (*Reln*) and brain-derived neurotrophic factor (*Bdnf*) [47, 48], which have been reported to be influenced by DNA methylation and neuronal activity [49-51]. Neither *Reln* nor *Bdnf* exon I and IX transcript levels were altered by MET and PrL DBS, whereas *Bdnf* exon IV transcript levels were significantly increased by MET alone and appeared to be synergistically enhanced by MET and PrL DBS (Fig. 2G). Given the associations of *Bdnf* exon IV expression with synaptic plasticity and contextual learning [50], we next evaluated the transcript levels of postsynaptic density protein 95 (*Psd95*), synaptophysin (*Syp*), and cAMP response element-binding protein (*Creb*). We found MET and PrL DBS enhanced the expressions of *Psd95* and *Creb*, but not *Syp* (Fig. 2H), suggesting that MET and PrL DBS may elicit a postsynaptic effect that contributes to the observed behavioral restoration in aged animals. To assess the neuronal activity changes in the hippocampus induced by PrL DBS, we examined the expression levels of immediate-early genes FBJ osteosarcoma oncogene (*c-fos*) and early growth response 1 (*Egr1*). We showed that PrL DBS could significantly increase the transcript levels of both genes. Interestingly, MET with PrL DBS further increased the transcript levels of these two genes, indicating that MET-induced DNA methylation and PrL DBS may work in concert to facilitate hippocampal activity and neuroplasticity (Fig. 2I). Taken together, our analysis of DNA methylation/demethylation- and plasticity-related genes revealed candidate genes whose altered expression possibly underlie the observed anxiolytic and memory-enhancing effects induced by PrL DBS.

**Figure 3.**
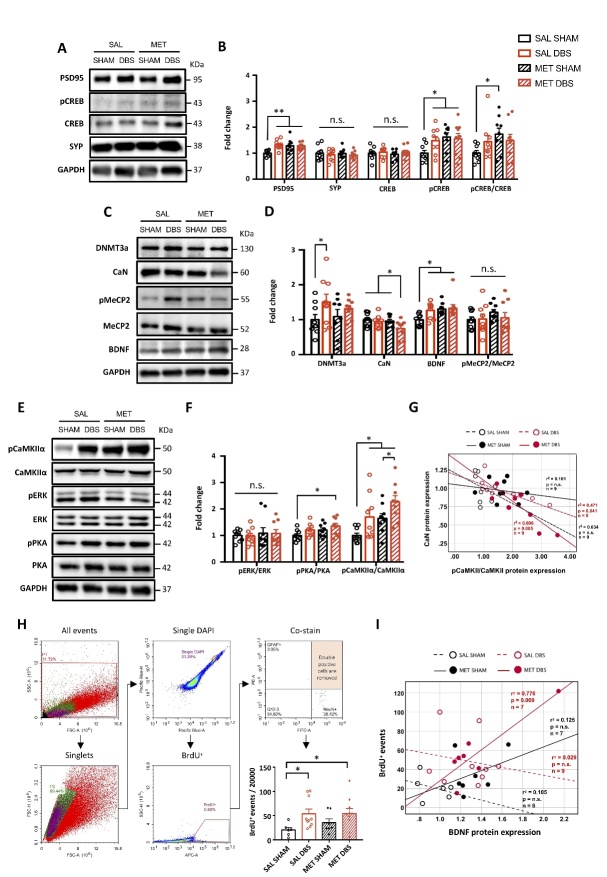
Protracted MET treatment and PrL DBS enhances neuroplasticity and hippocampal neurogenesis through the PKA-CaMKIIα-BDNF pathway. Representative Western blot images of neuroplasticity-associated proteins (A). Densitometric measurement by two-way ANOVA analysis of PSD95 (Treatment, p = 0.038; Stimulation, p = 0.01; Treatment x Stimulation, p = 0.01), SYP (p > 0.376), CREB (p > 0.369), pCREB (Treatment, p = 0.044) and pCREB/CREB ratio (Treatment, p = 0.074) showed enhanced neuroplasticity in MET and DBS animals (B). Representative Western blot images of proteins involved in memory neuroepigenetics (C). Two-way ANOVA revealed a significant stimulation effect (p = 0.041) for DNMT3a, insignificant treatment (p = 0.071) and significant stimulation (p = 0.041) effects for CaN, and significant treatment (p = 0.022) and insignificant stimulation (p = 0.068) effects for BDNF. No difference in the pMeCP2/MeCP2 ratio was observed among all groups (p > 0.205) (D). Representative Western blot images of proteins involved in the intermediate pathways of CREB phosphorylation (E). Two-way ANOVA showed no changes in pERK/ERK ratio (p > 0.499), significant changes in pPKA/PKA ratio (treatment, p = 0.048; stimulation, p = 0.046) and pCaMKIIα/CaMKIIα ratio (Treatment, p = 0.006; Stimulation, p = 0.004). (F). Scatter plot displaying significant correlations between CaN and pCaMKIIα/CaMKIIα ratio in SAL-DBS and MET-DBS animals, indicating that the inhibition of CaN by PrL DBS was strongly associated with the activation of CaMKIIα in the hippocampus. All groups: n=9. (G). Neurogenesis quantification by flow cytometry analysis for BrdU. Scatter plot displaying a selection of nuclei stained with DAPI, subsequent gating for BrdU, and removal of events doubly stained for GFAP and NeuN. Quantification of all groups revealed a significant main effect of stimulation (p = 0.012). More BrdU-positive events were found in SAL-DBS and MET-DBS animals compared to SAL-SHAM animals (H). Scatter plot displaying significant positive correlations between BrdU-positive cell count and BDNF protein expression in MET-DBS animals. SAL-SHAM: n=6; SAL-DBS: n=10; MET-SHAM: n=7; MET-DBS: n=10. (I). *, p<0.05; **, p<0.01; n.s., not significant. Data presented as mean ± s.e.m.

### MET and PrL DBS enhance neuroplasticity-related protein expressions and hippocampal neurogenesis

Based on the altered expression of memory-related genes induced by MET and PrL DBS, we next investigated the effects on the expression and activation of proteins involved in synaptic plasticity that possibly underlie the observed behavioral improvements shown in Figure 1. We demonstrated enhanced expression of PSD95 but unaltered levels of SYP in all treatment groups compared to the SAL-SHAM group, suggesting the effects of MET and PrL DBS maybe through a postsynaptic mechanism. We next measured the levels of CREB and its phosphorylated form (pCREB), a protein crucial for memory formation and synaptic plasticity [52]. We found the level of pCREB was enhanced in all treatment groups, while the level of total CREB remained unchanged (Fig. 3A, B). Given that CREB is a nuclear calcium signaling target [52], we assessed the expression levels of proteins affected by calcium signaling including DNMT3a, CaN, BDNF, and methyl-CpG binding protein 2 (MeCP2) in the hippocampus (Fig. 3C). We found that PrL DBS significantly enhanced the expression of DNMT3a, whereas both PrL DBS and MET increased the expression of BDNF. Interestingly, the MET-DBS group had the highest BDNF expression among all the groups. Moreover, CaN expression was decreased by MET and PrL DBS combined, but not separately. We next measured MeCP2, which is activated by phosphorylation and induces downstream *Bdnf* transcription [47, 49]. Surprisingly, neither MET nor PrL DBS altered MeCP2 activity or the pMeCP2/MeCP2 ratio, which excludes the role of MeCP2 in augmenting the BDNF expression due to MET and PrL DBS (Fig. 3D). It was reported that CREB can function as a downstream postsynaptic target molecule for the activation of extracellular signal-regulated kinase (ERK) [53], protein kinase A (PKA) [54], and calcium/calmodulin-dependent protein kinase IIα (CaMKIIα) [55]. We examined the effects of MET and PrL DBS separately or combined on the activity of these kinases, which showed a MET-dependent increase in PKA activity, enhanced CaMKIIα activity, but no change in ERK activity (Fig. 3F). Interestingly, MET-DBS animals showed the highest increases in both PKA and CaMKIIα activities, indicating a synergistic effect on the regulation of these two kinases. Given that both CaN and CaMKIIα are essential for synaptic strength [56], we performed a correlation study to examine the relationship between the expression of CaN and the activation of CaMKIIα. We observed a significant negative correlation between these two parameters in MET-DBS animals, suggesting an association between the enhanced activity of CaMKIIα and reduced expression of CaN under combined MET and PrL DBS (Fig, 3G). Our results, together with the findings from previous studies [45], demonstrate that CaN acts as an inhibitory constraint on PKA and CaMKIIα, which suggests the downregulation of CaN and simultaneous activation of PKA and CaMKIIα may collectively underlie the increased phosphorylation of CREB and enhanced expression of plasticity-related proteins induced by MET and PrL DBS. As neurogenesis is one of the mechanisms by which neuromodulation exerts its therapeutic effects and is also influenced by DNA methylation [6, 9, 10, 36, 57], we further explored the effects of MET and PrL DBS on cell proliferation. The flow cytometry analysis revealed remarkably increased numbers of BrdU-positive cells in the hippocampus of SAL-DBS and MET-DBS animals compared to SAL-SHAM animals, indicating enhanced hippocampal proliferation in the DBS animals (Fig. 3H). Given the association between BDNF and neurogenesis [41, 47, 58, 59], we also probed the potential role of BDNF underlying the effects of MET and PrL DBS on hippocampal cell proliferation. Interestingly, we observed a significant positive correlation between BDNF protein expression and BrdU-positive cell count under MET and PrL DBS, indicating their co-administration may promote neurogenesis via a BDNF-dependent mechanism (Fig. 3I).

**Figure 4.**
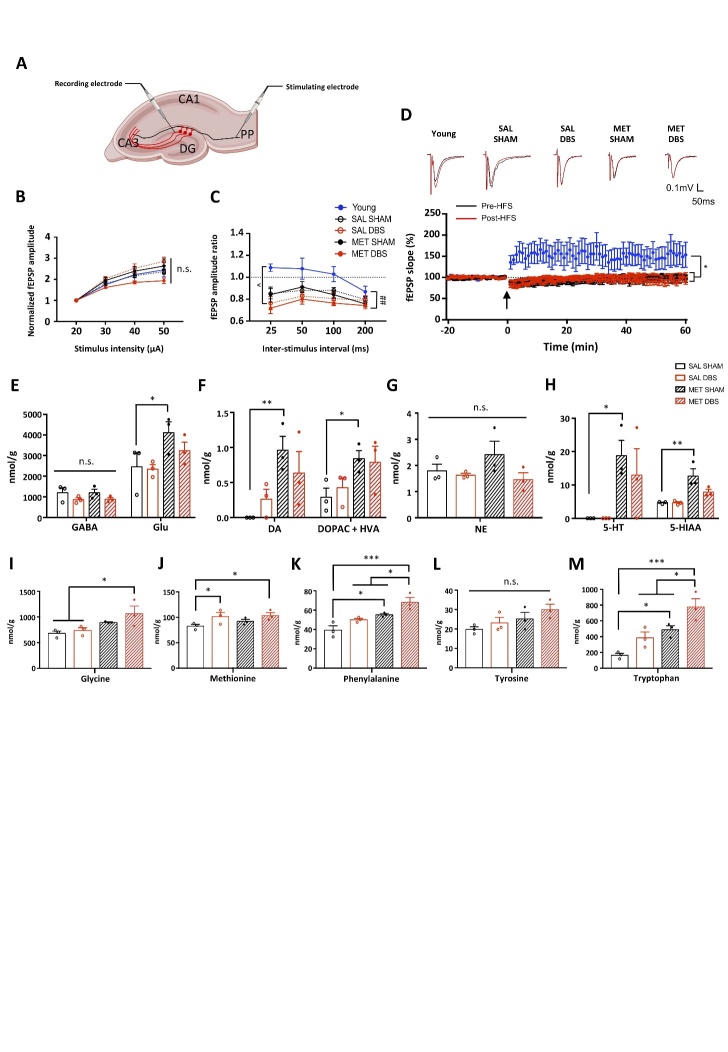
Protracted MET treatment and PrL DBS did not improve hippocampal synaptic plasticity, but MET modulates neurotransmitters and amino acids in the dorsal hippocampus. Schematic diagram showing the configuration for recording and placement of stimulating electrodes for examining the synaptic properties of perforant pathway (PP) projections to the dentate gyrus (DG) (A). The Input-output (I/O) function of the field EPSP slope in response to increasing stimulation intensity (Intensity x Group, p = n.s.) was analysed by two-way repeated measures ANOVA followed by Bonferroni-corrected pairwise comparison. (B). Short-term synaptic plasticity measured by paired-pulse ratio (peak amplitude of 1^st^ EPSP divided by 2^nd^ EPSP) at different stimulation intervals (Group, p = 0.013; Group x interval, p = 0.013). ^, p<0.05, Young vs. SAL-DBS; ##, p< 0.01, Young vs. MET-DBS (C). Long-term synaptic potentiation (LTP) at PP-DG synapses recorded with HFS delivered at time 0 (min). Aged rats showing impaired LTP compared to their young counterparts (Group, p<0.001; Time, p<0.001; Group x Time, p = 0.03). SAL-SHAM: n=6; SAL-DBS: n=6; MET-SHAM: n=8; MET-DBS: n=6; naïve young group: n=4. (D). MS analysis of various neurotransmitters and metabolites in the dorsal hippocampus with two-way ANOVA showing the level of gamma-aminobutyric acid (GABA) (p = n.s.), glutamate (Glu) (p = 0.031) (E), dopamine (DA) (p = 0.009), 3,4-Dihydroxyphenylacetic acid (DOPAC) and homovanillic acid (HVA) (p = 0.022) (F), norepinephrine (NE) (p = n.s.) (G), serotonin (5-HT) (Treatment, p = 0.008) and 5-hydroxyindole-3-acetic acid (5-HIAA) (Treatment, p = 0.001) (H). Levels of amino acids substantially involved in metabolism and neurotransmitter synthesis, including glycine (Treatment, p = 0.012) (I), methionine (Stimulation, p = 0.028) (J), phenylalanine (Treatment, p = 0.001; Stimulation, p = 0.01) (K), tyrosine (Treatment, p = 0.051) (L), and tryptophan (Treatment, p = 0.001; Stimulation, p = 0.007). SAL-SHAM: n=3 pools; SAL-DBS: n=3 pools; MET-SHAM: n=3 pools; MET-DBS: n=3 pools (3 animals per pool). (M). MS samples were randomly pooled in triplicate. *, p<0.05; **, p<0.01; n.s., not significant. Data presented as mean ± s.e.m.

### MET and PrL DBS do not significantly alter hippocampal synaptic plasticity, but induce widespread changes in neurotransmitter and amino acid levels

To investigate the effects of MET and PrL DBS on the perforant pathway-dentate gyrus (PP-DG) network, which is reported to be associated with memory functions, we performed PrL DBS with MET administration on a separate group of aged rats (n=26) to obtain ex vivo brain slices for electrophysiological recording (Fig. 4A). Analysis showed no significant differences in the intrinsic excitability of PP-DG among aged and young rats, as indicated by the synaptic input/output function (Fig. 4B). Paired-pulse response was not significantly altered in SAL-SHAM and MET-SHAM animals compared to young naïve animals. However, aged animals receiving PrL DBS showed inhibited paired-pulse response in the PP-DG network compared to young naïve group, as shown by a decreased paired-pulse ratio during the stimulus interval (Fig. 4C). This may imply a depletion of presynaptic vesicles during neurostimulation, which can inform the state of PP-DG network of aged animals administered PrL DBS. This observation was further extended to changes in long-term potentiation (LTP). High-frequency stimulation (HFS) in young rats reliably induced LTP in the PP-DG, whereas HFS in aged rats consistently resulted in significant depression (Fig. 4D). Our results resemble the findings in two previous studies on the blockade of LTP by infralimbic cortical stimulation and acute antidepressant treatment [60, 61], both of which also modulated endogenous serotonergic transmission in the hippocampus. To this end, we next asked whether the effects of MET and PrL DBS were mediated through the modulation of neurotransmitters related to learning and memory [62-64]. The mass spectrometry (MS) analysis of GABA, Glu, DA, DOPAC, HVA, NE, 5-HT, and 5-HIAA levels in the dissected dorsal hippocampus (dHPC) of aged rats[22] revealed that MET significantly augmented Glu, DA, DOPAC+HVA, 5-HT, and 5-HIAA levels without altering GABA and NE levels (Fig. 4E-H), which suggest the replenishment of MET facilitates neurotransmitter synthesis and metabolism. Further quantification of amino acids contributing to neurotransmitter synthesis supported these observations. We found that MET and PrL DBS increased levels of glycine and methionine, respectively (Fig. 4I, J). Moreover, MET treatment increased phenylalanine and tryptophan, which are precursors for DA and 5-HT, whereas co-administration with PrL DBS potentiated these increases (Fig. 4K, M), further supporting its synergistic effects with MET. In addition, MET treatment led to a marginally significant increase in tyrosine levels (Fig. 4L), implicating the involvement of the dopaminergic system. These findings suggest a potential mechanism of the memory-enhancing effects of MET and PrL DBS through the restoration of dopaminergic and serotonergic transmission.

### PrL DBS showed anxiolytic effect but failed to rescue the memory deficits induced by chronic RG108 administration

**Figure 5.**
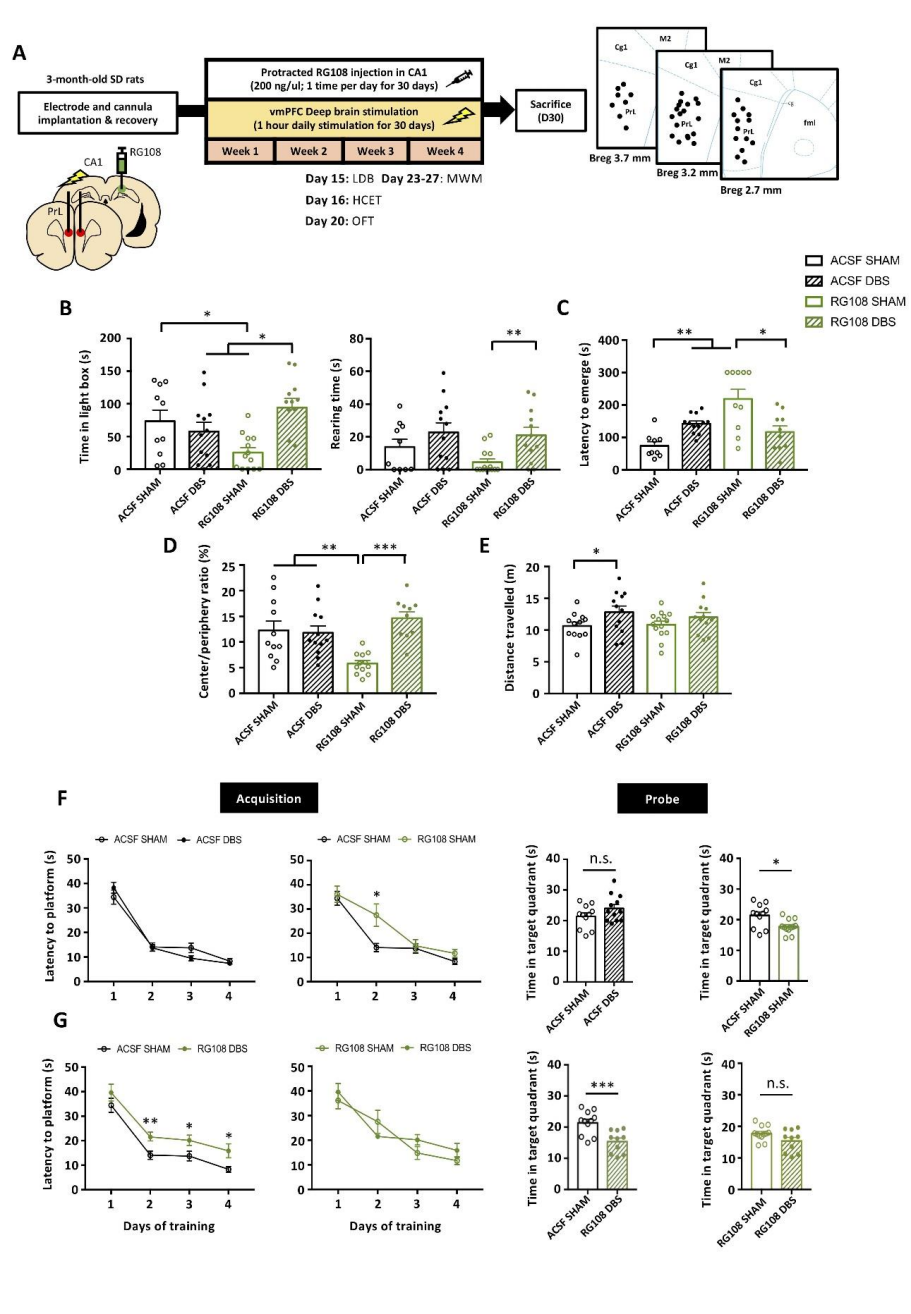
PrL DBS showed anxiolytic effect but failed to rescue the memory deficits induced by chronic RG108 administration. Schematic representation of the experimental timeline and behavioral tests for anxiety and memory. Animals were bilaterally implanted with a cannula in the hippocampal CA1 for chronic RG108 infusion and stimulating electrodes in the PrL (A). The data were analyzed by two-way ANOVA, followed by orthogonal planned comparison. RG108 administration significantly reduced time spent in light box, whereas PrL DBS significantly increased the time spent in the light box in RG108-infused animals (Stimulation, p = 0.048; Treatment x Stimulation, p = 0.002). PrL DBS had significant effects on rearing behavior in the light box (Stimulation, p = 0.006) (B). RG108 significantly increased the latency to emerge from home cage (Treatment, p = 0.003), whereas PrL DBS reversed this deficit (Treatment x Stimulation, p < 0.001), indicating DNMT inhibition does not affect the anxiolytic effects of PrL DBS (C). RG108 reduced the time spent in the center of the open field, which was restored by PrL DBS (Treatment x Stimulation, p < 0.001) (D). PrL DBS increased the locomotor activity of the animals (Stimulation, p = 0.026) (E). Two-way ANOVA with repeated measures revealed significant treatment effect (p < 0.001) during training days in the MWM task. No significant effect was observed in the interaction between RG108 and PrL DBS (Treatment x Stimulation, p = n.s.), indicating that PrL DBS failed to rescue the learning deficit induced by RG108. Both RG108-SHAM and RG108-DBS animals took longer to locate the hidden platform during training (F) and spent significantly less time in the target quadrant (Treatment, p < 0.001). ACSF-SHAM: n=12; ACSF-DBS: n=12; RG108-SHAM: n=14; RG108-DBS: n=12. (G) compared to ACSF-SHAM animals. * p<0.05; ** p<0.01; ***, p<0.001; n.s., not significant. Data presented as mean ± s.e.m.

Given the association of *Dnmt3a* with plasticity-related genes and its synergistic upregulation by MET and PrL DBS in the hippocampus of aged animals, we examined if the inhibition of hippocampal DNMT activity in young adult animals with intact cognition would produce phenotypical deficits resembling aged animals. We also examined hippocampal DNMT as a potential mechanism underlying the therapeutic effects of PrL DBS. We inhibited hippocampal DNMT activity by administering a localized infusion of a potent DNMT inhibitor RG108 in young animals receiving chronic PrL DBS (Fig. 5A). We found the infusion of RG108 resulted in anxiogenic behaviors in the LDB test, as indicated by reduced rearing behavior and time spent in the light box (Fig. 5B). These findings were substantiated in RG108-SHAM animals in the HCET, as indicated by a prolonged latency to emerge from the home cage (Fig. 5C). Although PrL DBS restored the deficits observed in the LDB test and HCET in RG108-infused animals, it exerted minimal effects on ACSF-infused animals. In the OFT, RG108-SHAM animals spent significantly less time in the center of the open field, indicating anxiogenic behavior, whereas PrL DBS effectively restored this behavior. In ACSF-infused animals, PrL DBS did not affect the time spent in the center in open field (Fig. 5D). The administration of RG108 did not alter the locomotor activity of the animals, as shown by the similar distances traveled in the open field, whereas ACSF treatment resulted in a small significant increase in the distance traveled by ACSF-DBS animals (Fig. 5E). Overall, these findings confirmed that the anxiolytic effects of PrL DBS were not dependent on hippocampal DNMT activity. We next assessed whether inhibition of hippocampal DNMT activity could alter the cognitive-enhancing effects of PrL DBS. The MWM task showed both RG108-SHAM and RG108-DBS animals took longer to locate the hidden platform during training (Fig. 5F) and spent significantly less time in the target quadrant during probe testing (Fig. 5G). Again, there were no observed differences in spatial learning and memory between ACSF-SHAM and ACSF-DBS animals, probably due to a ceiling effect of cognition in ACSF-SHAM animals. Taken together, our findings suggest that the memory-enhancing effects of PrL DBS may be dependent on hippocampal DNMT activity, whereas its anxiolytic effects may be mediated by other DNMT-independent mechanisms in the hippocampus.

### PrL DBS partially rescues the molecular deficits induced by hippocampal DNMT inhibition.

**Figure 6.**
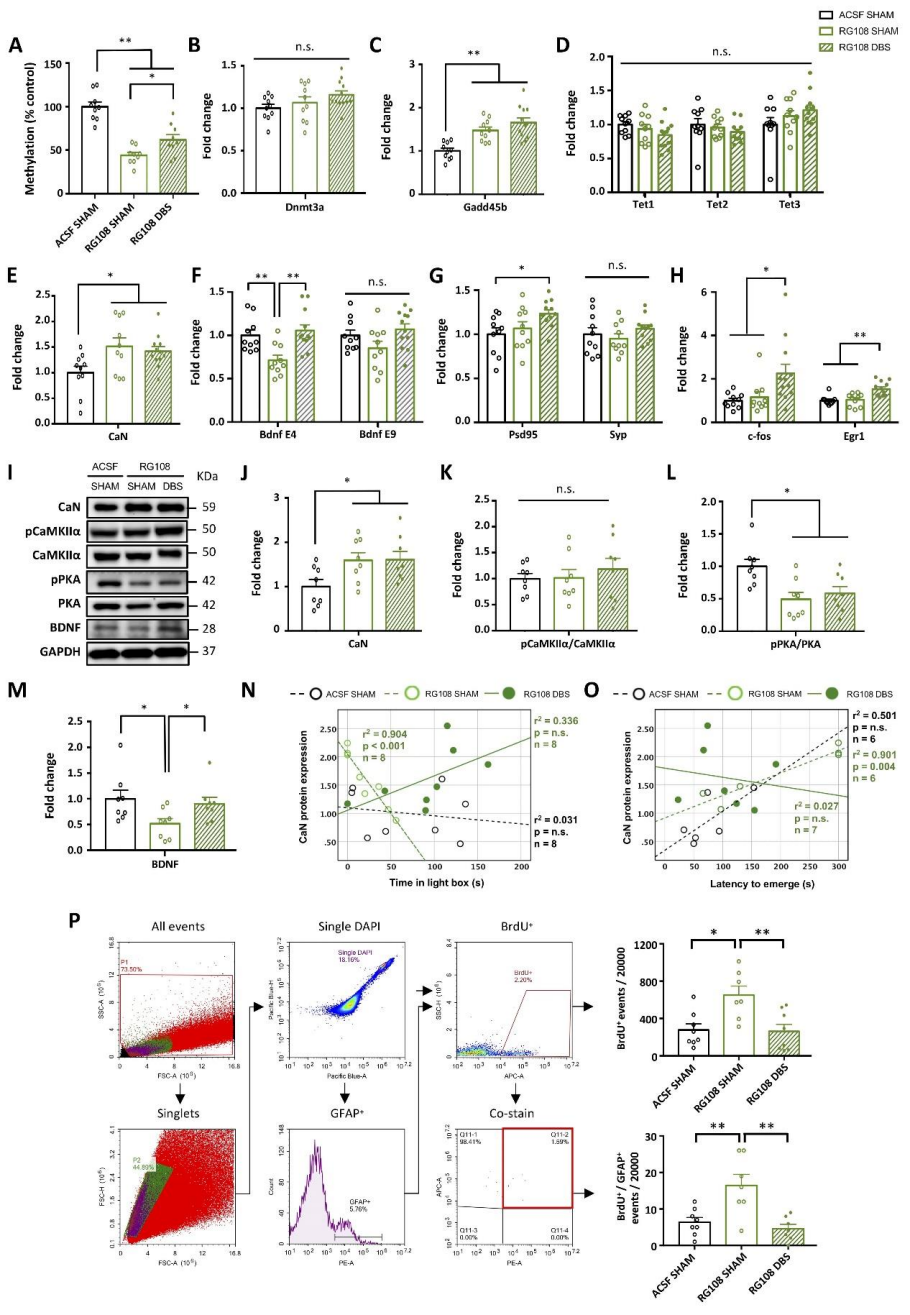
Hippocampal DNMT inhibition elevates CaN level, suppresses the PKA-CaMKIIα pathway, and induces astrogliosis. One-way ANOVA analysis revealed that RG108-infused animals showed decreased global DNA methylation compared to ACSF-infused control animals, which was partially rescued by PrL DBS (p < 0.001) (A). Quantitative reverse-transcription PCR analysis of *Dnmt3a* (p = n.s.) (B), *Gadd45b* (p < 0.001) (C), *Tet1, Tet2*, and *Tet3* (p > 0.153) (D), *CaN* (p = 0.022) (E), *Bdnf* exon IV (p =0.001) and exon IX (p = 0.079) (F), *Psd95* (p = 0.032) and *Syp* (p = 0.474) (G), and *c-fos* (p = 0.013) and *Egr1* (p < 0.001) (H). Representative Western blot images of neuroplasticity-associated proteins (I). Densitometric analysis of CaN (p = 0.033) (J), pCaMKIIα/CaMKIIα (p = n.s.) (K) pPKA/PKA (p = 0.007) (L) and BDNF (p = 0.048). (M). Scatter plot by Pearson correlational analysis of the RG108-SHAM group showing the expression of CaN protein was negatively correlated with the time spent in the light box (N) and was positively correlated with the latency to emerge from the home cage (O), indicating CaN plays a role in RG108-induced anxiety. Neurogenesis quantification by flow cytometry analysis of BrdU. Scatter plot displaying a selection of nuclei stained with DAPI and subsequent gating for BrdU and GFAP. Quantification of all groups revealed a significant main effect in both BrdU^+^ (p = 0.004) and BrdU^+^/GFAP^+^ co-staining (p < 0.001). All groups: n=8. (P). *, p<0.05; **, p<0.01; n.s., not significant. Data presented as mean ± s.e.m.

**Figure 7.**
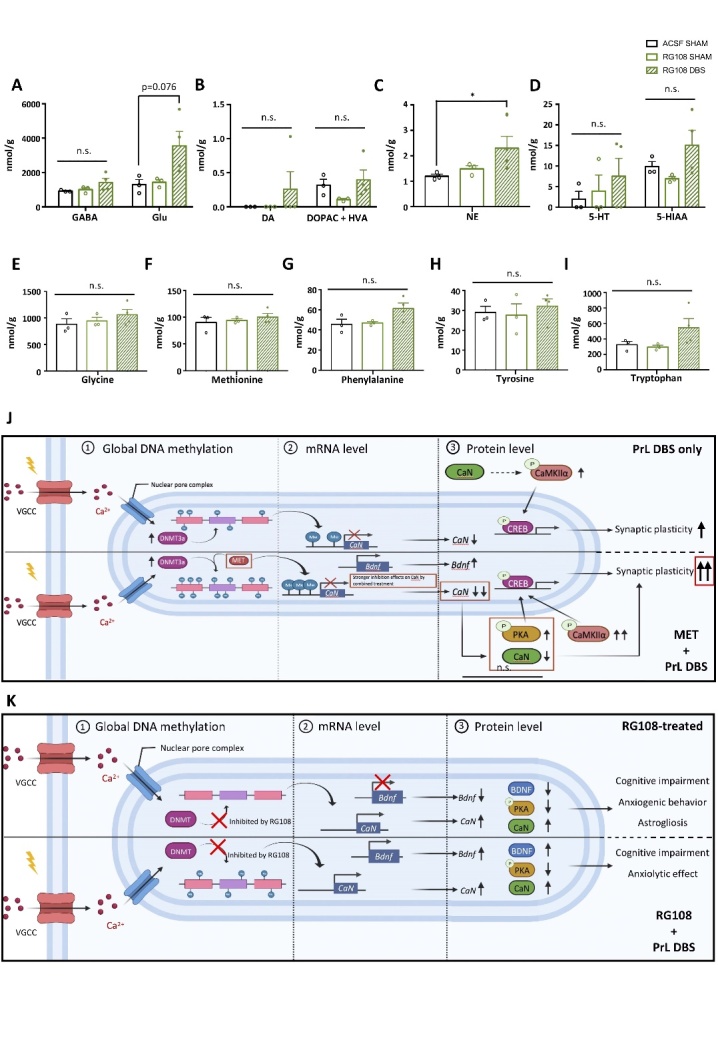
Hippocampal DNMT inhibition abolishes changes in neurotransmitter and amino acid levels involved in dopaminergic and serotonergic systems. MS analysis of various neurotransmitters and metabolites in the dorsal hippocampus showed levels of gamma-aminobutyric acid (GABA) (p = n.s.), glutamate (Glu) (p = 0.038) (A), dopamine (DA) (p = n.s.), 3,4-dihydroxyphenylacetic acid (DOPAC) and homovanillic acid (HVA) (p = n.s.) (B), norepinephrine (NE) (p = 0.049) (C), and serotonin (5-HT) (p = n.s.) and 5-hydroxyindole-3-acetic acid (5-HIAA) (p = n.s.) (D). Levels of amino acids substantially associated with the metabolism and synthesis of neurotransmitters included glycine (p = n.s.) (E), methionine (p = n.s.) (F), phenylalanine (p = n.s.) (G), tyrosine (p = n.s.) (H), and tryptophan (p = n.s.) (I). ACSF-SHAM: n=3 pools; RG108-SHAM: n=3 pools; RG108-DBS: n=4 pools (3 animals per pool). MS samples were randomly paired in triplicate or quadruplicate. The data were analyzed by non-parametric Kruskal-Wallis test followed by pairwise comparison. Proposed model of DNA methylation-dependent enhancement of synaptic plasticity by PrL DBS (J). Activation of voltage-gated calcium channel by PrL DBS induced Ca^2+^ influx, which activated the transcription of *Dnmt3a* and its subsequent protein expression. Such a cellular response enhanced the capacity of *de novo* DNA methylation, which was further expanded by the MET treatment. The increased DNA methylation in turn altered the transcription of memory-related genes (*CaN* and *Bdnf* exon IV) and subsequent protein cascades (PKA, CREB, and CaMKIIα phosphorylation). Dual treatment of MET and PrL DBS exerted a stronger inhibitory effect on CaN expression, which possibly induced more robust activation of PKA and CaMKIIα (A). Meanwhile, inhibition of DNMT activity by RG108 resulted in global DNA hypomethylation and reduced *Bdnf* exon IV transcription. PrL DBS partially restored the DNA methylation level and rescued *Bdnf* exon IV transcription. RG108 also resulted in increased CaN transcript and protein levels, which may contribute to behavioral deficits and astrogliosis (B). This proposed model was created in BioRender.

We next examined how the molecular changes elicited by intrahippocampal RG108 infusion could induce the observed behavioral deficits and abolish the memory-enhancing effects of PrL DBS. We assessed the gene and protein expression changes due to RG108 administration on the MET and PrL DBS treatment. We demonstrated that RG108 infusion significantly reduced the DNA methylation level in the hippocampus of young animals, which was partially rescued by PrL DBS (Fig. 6A). Further analysis of the transcript levels in the DNA methylation machinery revealed neither RG108 infusion nor PrL DBS altered the expression levels of hippocampal *Dnmt3a* and *Tets* (Fig. 6B, D). Interestingly, both RG108-SHAM and RG108-DBS animals showed increased transcript levels of hippocampal *Gadd45b* and *CaN* (Fig. 6C, E). Epigenetic modification has been shown to substantially alter *Bdnf* transcription, thereby affecting memory [50]. Indeed, RG108 infusion resulted in a marked decrease of *Bdnf* exon IV mRNA, which was effectively rescued by PrL DBS (Fig. 6F). Given the critical role of BDNF in neuroplasticity [47], we investigated whether deficient *Bdnf* exon expression induced by RG108 would affect the expression of plasticity-related genes. We found that RG108 did not alter the expression of *Psd95* or *Syp* mRNA, whereas PrL DBS specifically increased the expression of *Psd95*, substantiating a postsynaptic mechanism (Fig. 6G). Moreover, RG108 did not affect the activation of the hippocampus by PrL DBS, as indicated by significantly augmented expressions of *c-fos* and *Egr1* in RG108-DBS animals (Fig. 6H). We assessed the levels of proteins altered by MET and PrL DBS in our previous experiment (Fig. 6I). We found both RG108-SHAM and RG108-DBS animals had elevated CaN protein levels, corroborating the results of the gene assay (Fig. 6J). Meanwhile, RG108 blocked CaMKIIα and PKA activation in DBS animals (Fig. 6K, L). Notably, PrL DBS was able to rescue the reduced BDNF protein expression induced by RG108 (Fig. 6M). Correlation studies showed CaN protein levels were significantly correlated with the anxiogenic behaviors exhibited by RG108-SHAM animals (Fig. 6N, O), indicating a potential role of CaN in anxiety. However, the anxiolytic activity of PrL DBS may be exerted via a CaN-independent mechanism, as no significant correlations were found between CaN protein level and the behavioral parameters in RG108-DBS animals. Based on previous studies that implicated CaN in astrogliosis during the aging process [44, 65, 66], we assessed the expressions of BrdU and glial fibrillary acidic protein (GFAP) in the hippocampus. Flow cytometry analysis showed increased BrdU-positive cells in the hippocampus of RG108-SHAM animals, indicating the intrahippocampal infusion of RG108 stimulated neurogenesis, which could be restored by PrL DBS to levels comparable to that of control animals. Further analysis of newly generated neuronal cells revealed a high number of cells double-labeled for BrdU and GFAP in RG108-SHAM animals, which was reversed by PrL DBS (Fig. 6P), substantiating a role of DNA demethylation in astrogliosis. Further MS analysis of hippocampal neurotransmitters and amino acids showed enhanced Glu and NE levels in RG108-DBS animals, but no changes in GABA, DA, DOPAC+HVA, 5-HT, 5-HIAA, glycine, methionine, phenylalanine, tyrosine, and tryptophan levels (Fig. 7A-I). In contrast to studies that reported vmPFC DBS increased hippocampal 5-HT [67], inhibition of hippocampal DNMT did not lead to altered 5-HT levels. These findings may suggest that hippocampal DNMT activity contributes to both dopaminergic and serotonergic transmission. Collectively, our results suggest that hippocampal DNMT inhibition can induce cognitive impairments by promoting DNA demethylation, leading to the inactivation of plasticity-related pathways and elevated astrogliosis resembling aging [41], and by abolishing the effects of PrL DBS on serotonergic and dopaminergic systems and cognition.

## DISCUSSION

Our previous studies established the PrL region as a target for DBS to effectively attenuate age-related cognitive impairments [6, 8]. Recent studies on neuroepigenetics have identified molecular targets that may hold promise for therapies that enhance cognition [11]. Furthermore, in aged animals, hippocampal DNMT and global DNA methylation are both decreased, and have been shown to correlate with cognitive deficits [15]. Based on these observations, several preclinical studies have investigated the therapeutic potential of methyl supplements for modulating DNA methylation, which showed they could improve cognition in diseases such as temporal lobe epilepsy [68] and Alzheimer’s disease (AD) [69]. Our study aimed to test the hypothesis that PrL DBS administered together with MET can ameliorate aged-related cognitive dysfunction via a DNA methylation-dependent mechanism. We showed that PrL DBS with MET treatment elicited more robust memory rescue in our aged animal model with cognitive impairment. We found that PrL DBS alone only improved spatial learning but not memory retrieval in aged animals, which supports previous clinical work that reported a lower therapeutic efficacy of neuromodulation associated with increased biological age[70]. More importantly, we demonstrated MET and PrL DBS had potential synergistic effects on enhancing cognition in aged animals through restoring the DNA methylation level.

We selected the dHPC as the region of interest because of its function in spatial memory encoding and its implicated role on the effects of PrL DBS[6, 8, 22]. Our study showed that MET not only restored global DNA hypomethylation in the dHPC, but together with PrL DBS, also synergistically increased DNA methylation in aged animals. A previous study demonstrated MET could enhance DNA methylation in the hippocampus by facilitating the production of S-adenosylmethionine (SAM) [68]. Our study showed that PrL DBS upregulated hippocampal *Dnmt3a*, which has recently been reported to possess immediate early gene properties [15]. This may suggest a greater demand for methyl group turnover by DNMT3a under increased neuronal activity, thereby leading to enhanced *de novo* DNA methylation. Notably, recent studies revealed that neuromodulation techniques are capable of inducing widespread methylome changes. These changes encompass differential methylation patterns in gene promoters and bodies, revealing bidirectional transcription outcomes [13, 36, 42, 71-73]. Previous research reported differentially methylated genes in the formation of effective memory [74]. Here, we demonstrated that MET treatment facilitated the effects of PrL DBS on enhancing the expression of memory-related genes *Bdnf* exon IV, *c-fos*, and *Egr1*, which have reduced transcriptional efficiency in aged animals [75-77], while simultaneously inhibiting the memory-suppressing gene *CaN*. Furthermore, besides reduced CaN expression, MET-DBS animals showed enhanced CaMKIIα and PKA phosphorylation and increased BDNF expression, suggesting enhanced plasticity. Surprisingly, we found a depressed paired-pulse response in the PP-DG network of aged rats receiving PrL DBS, considering that these animals exhibited memory improvements and antidepressant effects in the behavioral assays. One explanation could be that the neurotransmitter pools were depleted after 1 h of PrL DBS by the time the brain slices were subjected to HFS for electrophysiological recording, given our previous finding [6, 8] and current results demonstrated hippocampal activation upon PrL stimulation. It is important to consider the neuro-physiological changes in the hippocampus throughout the DBS treatment. In a study by Hescham et al., they demonstrated that the hippocampal acetylcholine levels peaked after 20 min of fornix stimulation before declining back to its basal level [78]. Notably, differential responses in distinct neurotransmission systems were reported upon hippocampal activation. For instance, Hamani et al. observed a prolonged increase in hippocampal 5-HT even after the 1-h vmPFC stimulation had ceased [67]. Overall, electrical stimulation of the above two brain targets was shown to enhance hippocampal neural activity. It is worth noting that the animals in our study received 30-min stimulation prior to the behavioral assays, as we previously found this led to greater memory enhancements compared to no stimulation before testing [6]. Moreover, prolonged HFS may induce presynaptic depression due to reduced calcium entry [79], which may also lend support to our current findings. Consistent with previous reports in aged animals [80, 81], we observed no LTP in the PP-DG network, and both MET and PrL DBS did not alter this observation. This may be due to reduced expression of hippocampal NMDA receptor subunits NR1 and NR2B, as reported in aged rodents[82]. In fact, such age-related decline in the neurotransmission system may be the limiting factor on the electrophysiological responses observed in aged animals, despite the enriched neurotransmitter pool as shown in GC/MS measurements. Moreover, although the LTP blockade may resemble the effects of antidepressant administration, as mentioned above[61], this finding seemingly contradicts the spatial memory improvements indicated by the behavioral and molecular data. It should be noted that the whole of the dorsal hippocampus was microdissected out for the biochemical assays. Hence, it is possible that other subregions, such as CA1 and CA3, contributed to the molecular profile. Indeed, other pathways were previously shown to play a major role in memory. For example, distinct roles of the perforant pathway and the Schaffer collateral pathway were suggested to mediate memory encoding (<24 h retention) and retrieval (>24 h retention) respectively, with CA3 and DG lesions disrupting memory encoding and CA1 lesions producing deficits in retrieval [83, 84]. Further studies are needed to elucidate the time-dependent changes in synaptic plasticity as well as to delineate the involvement of specific pathways to explain the aforementioned findings accompanying memory enhancements induced by DBS.

We previously reported PrL DBS modulated monoamine neurotransmitters in the hippocampus [8, 22]. To probe the chronic effects of MET and PrL DBS on neurotransmission, we performed GC/MS to measure the levels of neurotransmitters and their corresponding metabolites. We found MET significantly increased levels of DA and 5-HT and their corresponding metabolites, which implies that MET can facilitate the production and metabolism of these neurotransmitters. The levels of both DA and 5-HT in MET-treated animals were well above the detectable thresholds compared to undetectable DA levels in the SAL-SHAM group and 5-HT levels in SAL-SHAM and SAL-DBS groups, which demonstrated that MET indeed enhanced DA and 5-HT levels. Congruently, our previous work also reported the involvement of monoamines in the memory-enhancing effects of PrL DBS[8]. This was further supported by our findings of widespread changes in amino acid levels elicited by MET and/or PrL DBS. Notably, MET specifically potentiated the levels of phenylalanine and tryptophan, which are precursors of DA and 5-HT, respectively. The MET-DBS animals exhibited the highest increase in phenylalanine and tryptophan levels, possibly indicating a higher production rate of the corresponding neurotransmitters. As SAM is also required for the synthesis of monoamines [85], these results may indicate that MET replenishment promotes monoamine production and together with PrL DBS enables their release and metabolism. Consistent with the study by Hamani et al. [67], we found no significant differences in hippocampal NE levels. Moreover, although no significant changes in GABA levels were observed, we found that MET enhanced hippocampal Glu levels. This may imply that the underlying mechanism of the memory enhancement may involve tuning the excitatory-inhibitory balance in the hippocampus. Indeed, shifting the balance towards excitation by selectively disrupting GABAergic neurotransmission results in enhanced memory performance [86]. Although this may be associated with memory function improvements as shown in the behavioral data, this seemingly contradicts the electrophysiology results. Nonetheless, it should be noted that the GC/MS method used in this study only measured total Glu, and such measurements do not necessarily reflect the rate of synaptic vesicle release, as this is subject to a number of factors including presynaptic calcium signaling and the availability of a readily reusable pool [87]. In addition, other sources of Glu release should also be considered, such as from astrocytes, which may also play an active role in the mechanism of DBS [88]. Indeed, we observed astrogliosis induced by chronic RG108 infusion, whereas PrL DBS suppressed the number of BrdU^+^/GFAP^+^ cells. The mechanism is possibly mediated through modulating the DNA methylation machinery that governs astrocyte differentiation, as a number of astrocytic genes were found to be demethylated prior to its differentiation. Further studies are needed to fully elucidate their contributions to the effects of DBS. Furthermore, we observed modest increases in glycine levels in MET-DBS animals and methionine levels in DBS animals. Both amino acids were previously demonstrated to possess antioxidant properties [89, 90]. It has been widely reported that DBS can confer neuroprotection in animal models of neurodegeneration induced by oxidative stress, such as animal models of Parkinson’s disease [91, 92], but the mechanism behind such rescue is unclear. Our results shed light on the possibility that modulation of amino acids by DBS may underlie these antioxidative effects, although this will require further testing.

Mechanistically, the failure of PrL DBS to rescue cognitive deficits in RG108-infused animals, despite the unaffected anxiolytic effects, suggests that hippocampal DNMT activity specifically drives the cognitive enhancements by PrL DBS. How PrL DBS drives anxiolytic behavior induced by RG108 remains to be investigated. Interestingly, we found an increased time spent in light box in ACSF Sham group (Fig. 5B) compared to young naïve rats (Fig. 1B), indicating an anxiogenic-like effect by electrode implantation in the absence of electrical stimulation. It is thought to be mediated by the transient inflammatory response at the implant site during the post-surgical period [93]. Nonetheless, we believe that this does not alter our interpretation of the results, as all animals (except for the young naïve animals in Fig.1) underwent identical surgical implantation procedures to allow fair comparison of the effects of DBS and RG108.

The results from the RG108 infusion experiments support that the molecular changes in PrL DBS, such as inhibition of CaN and activation of the PKA-CaMKIIα-BDNF signaling pathway, are dependent on DNMT and its downstream activation (Fig. 7J, K). These data are in line with previous work demonstrating that DNA methylation is necessary for neuroplasticity governing the response to memory retrieval [94]. Given the involvement of distinct DNA methylation in engram stability [95], we believe our findings also open up new avenues for targeting memory engram modulation in future studies [96].

Acute PrL stimulation performed at specific memory stages was shown to interfere with memory [22, 97], whereas memory retrieval was strengthened when it was administered chronically [6, 8, 98]. It is plausible that distinct DNA methylation patterns induced by different treatment modes of PrL DBS can contribute to transcriptional alterations that underly such paradoxical outcomes [98]. It would be interesting to examine the mechanisms that dictate the divergent consequences of neuromodulation. Interestingly, RG108 abolished most of the changes to the neurotransmitters and amino acids mentioned above, except Glu and NE, both of which were present at higher levels in RG108-DBS animals. Moreover, aged animals showed unaltered Glu and NE levels upon PrL DBS, which might be explained by age-dependent decline of the glutamatergic system and reduced NE input to the hippocampus [99-101]. Our findings also showed that DNMT inhibition, specifically in the dHPC, was sufficient to induce anxiogenic behavior and cognitive deficits resembling aging. This result emphasizes the integral role of DNA methylation in the aging process. Alzheimer’s disease is a prominent example of a neurodegenerative disorder harboring altered DNA methylation, in which accelerated DNA hypomethylation is observed [11, 12, 51, 102]. Indeed, preclinical work has demonstrated the efficacy of chronic SAM treatments in alleviating amyloid pathology through modulating DNA methylation[69]. Currently, DBS of the fornix, a region that regulates hippocampal activity, has received promising feedback from clinical trials assessing its effects on AD [103, 104]. It remains to be seen whether DNA methylation plays a role in neuromodulation modalities for treating deficits in AD or other memory disorders [12, 105-107].

Taken together, this study expands on the therapeutic efficacy of PrL DBS by showing that hippocampal DNMT may be a potential therapeutic target through which PrL DBS exerts its memory-enhancing effects. We demonstrated for the first time the synergistic effects of MET supplementation and neuromodulation on increasing the DNA methylation level, activating plasticity-related pathways, as well as enhancing the dopaminergic and serotonergic neurotransmission, to strengthen the memory-enhancing effects of PrL DBS. We extended these results to young animals and established a role of hippocampal DNMT activity in the memory-enhancing effects of PrL DBS. We also identified a DNA methylation-dependent mechanism underlying the cognitive enhancements by PrL DBS. Lastly, our findings highlight potential crosstalk between pharmacological interventions targeting DNA methylation and neuromodulation techniques that may influence hippocampal plasticity to yield optimal effects on cognition. In this respect, ethical issues on modulation of memory function is also important to further enhance international collaboration of interdisciplinary research [4, 108] on clinical applications for both invasive and non-invasive brain stimulation therapy [27, 109, 110].

## Data Availability

The datasets used and/or analyzed during the current study are available from the corresponding author on reasonable request.
